# ANCA-associated pauci-immune glomerulonephritis in a patient with bacterial endocarditis: a challenging clinical dilemma 

**DOI:** 10.5414/CNCS109076

**Published:** 2017-04-26

**Authors:** Andrea Cervi, Dylan Kelly, Iakovina Alexopoulou, Nader Khalidi

**Affiliations:** 1 Internal Medicine Residency Program,; 2 Department of Pathology and Molecular Medicine, and; 3 Department of Rheumatology, McMaster University, Hamilton, ON, Canada

**Keywords:** anti-neutrophil cytoplasmic antibodies, infective endocarditis, glomerulonephritis, immunosuppression

## Abstract

Purpose: We report the case of a 59-year-old man with chronic hepatitis B and C infection presenting with acute kidney injury and enterococcus faecalis-infective endocarditis (IE). An elevated proteinase-3 (PR3)-ANCA and pauci-immune glomerulonephritis (GN) on renal biopsy were discovered, corresponding to ANCA-mediated GN. We conducted a literature review to assess the role of ANCA in IE and treatment implications. Methods: On systematic review of the literature, we found five previous cases whereby IE caused by streptococcus and bartonella species were related to ANCA vasculitis-associated GN. Results: Most reports of IE-related GN are mediated by immune complex deposition and resolve following microbial clearance. Of the 5 cases of ANCA GN in the setting of IE, all had markedly elevated levels of PR3-ANCA with either a subacute or chronic course of infection. Patients were treated with a combination of steroids and cyclophosphamide (2/5), steroids and antibiotics alone (1/5), or with valvular replacement (2/5). Renal function was recovered in 4/5 patients. Conclusion: Infection is a major etiologic player in the formation of ANCA; however, the role of PR3-ANCA in IE remains unclear. Kidney biopsy is essential in differentiating IE-related GN due to infection and immune complex deposition versus ANCA-associated vasculitis. A paucity of reports on the development of GN in IE-associated ANCA vasculitis exists, highlighting the rarity of our case and lack of clear therapeutic strategies in a patient with active infection requiring immunosuppression. In this case, the patient’s chronic hepatitis B and C coinfection presented a unique challenge.

## Introduction 

Various patterns of kidney injury have been described during the course of infective endocarditis (IE) including renal cortical necrosis in the event of septic emboli or thrombotic microangiopathy, acute interstitial nephritis, and tubular necrosis as a consequence of drugs, bacterial toxins, or intravascular volume depletion [1]. Glomerulonephritis (GN) represents a unique form of renal injury that might also complicate the course of IE [2], typically through circulating immune complex deposition within glomeruli. 

Subacute and chronic inflammatory states including infection may also induce formation of ANCAs [4, 5] that may subsequently result in blood vessel damage leading to manifestations of vasculitis [6, 7]. Unlike immune complex-mediated GN in which renal recovery parallels resolution of infection [8], pauci-immune GN requires immunosuppression, posing a therapeutic challenge in the setting of bacterial sepsis. We report the case of a patient who developed proteinase-3 (PR3)-ANCA associated pauci-immune GN during the course of IE. In addition, we performed a systematic review and summary of similar cases described in the literature. 

## Case summary 

A 59-year-old man presented to the Emergency Department (ED) with a 6-month history of progressive low back pain, weight loss, and acute kidney injury (creatinine 418 µmol/L). Past medical history was significant for non-ischemic dilated cardiomyopathy and chronic left bundle branch block for which the patient had a prophylactic biventricular implantable cardioverter defibrillator (ICD). As well, he was found to be hepatitis-C positive 14 years earlier secondary to remote tattooing, with no evidence of liver fibrosis. The patient had also tested positive for the presence of anti-hepatitis B core antibodies but was negative for anti-hepatitis B core IgM and anti-hepatitis B surface antibodies, consistent with chronic inactive hepatitis B. He denied any current or previous recreational drug use, including cocaine, and sexual activity following separation from his wife several years earlier. 

The patient underwent a CT scan of his spine in the ED that revealed chronic discitis and osteomyelitis of his lower thoracic spine. The Orthopedic Surgery Service did not feel there was a role for surgery given the chronicity of the disease; consequently, the patient was admitted to Internal Medicine and was empirically treated for vertebral osteomyelitis. Blood cultures returned positive for *Enterococcus faecalis* that was treated with ampicillin and ceftriaxone, the latter for synergistic activity for treatment of possible IE pending results of an echocardiogram. Initial transthoracic echocardiogram revealed a 1.5 × 1 cm mass on the anterior tricuspid valve leaflet along with a 1 × 0.5 cm echo-dense mobile mass on the ICD wire of the right atrial side of his tricuspid valve. The ICD was later extracted, and the left ventricle (LV) lead was tunneled to the right side. 

**Figure 1.**
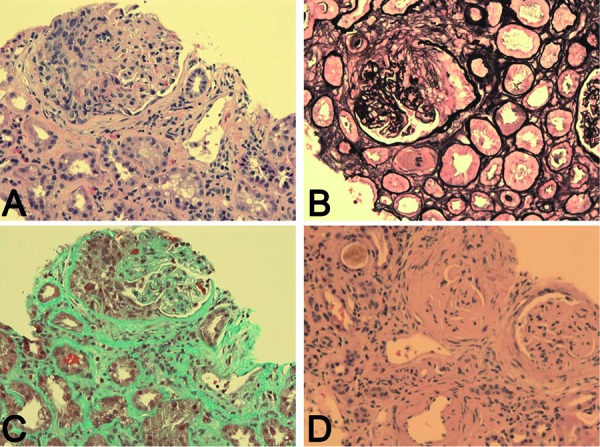
Renal biopsy histology demonstrating focal segmental proliferative and necrotizing glomerulonephritis. A: Glomerulus with segmental proliferation and crescent (hematoxylin-eosin stain, ×200). B: Glomerulus with segmental necrosis and crescent (Jones silver stain, ×200). C: Glomerulus with cellular crescent and interstitial fibrosis (Masson trichrome stain, ×200). D: Two glomeruli with fibro-cellular lesions and scarring indicative of chronicity (hematoxylin-eosin stain, ×200).

**Figure 2.**
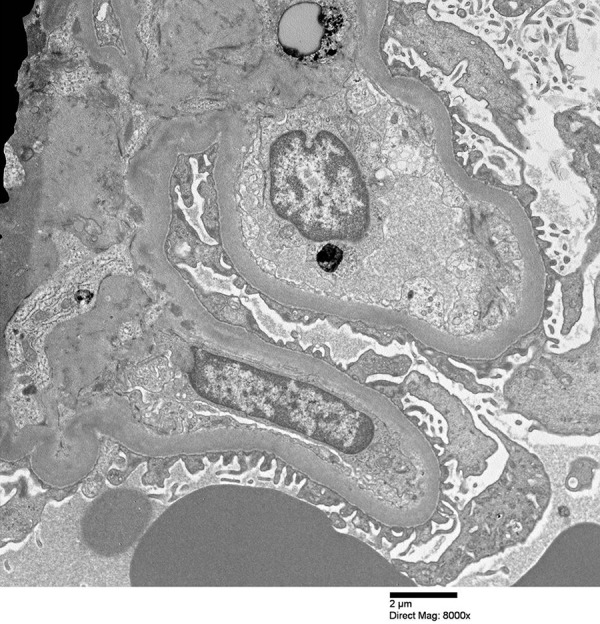
Part of glomerulus with focal fusion of foot processes (arrow) and absence of immune complexes (electron microscopy, ×8,000).

The patient’s creatinine continued to rise during his admission (peak 720 µmol/L) and was accompanied by microscopic hematuria and proteinuria on urinalysis. Consequently, the possibility of GN was entertained. A low C3 (0.5 g/L) and C4 (0.07 g/L) and strongly positive PR3-ANCA (> 8.0 AI) with a negative p-ANCA prompted renal biopsy that revealed focal segmental proliferative and necrotizing glomerulonephritis affecting > 50% of glomeruli with cellular and fibrocellular crescents and some segmental sclerosis (Figure 1), indicative of chronicity. Immunofluorescence and electron microscopy demonstrated a paucity of any significant immune complexes (Figure 2), and, given the severity and extent of necrotizing lesions, a diagnosis of pauci-immune GN consistent with ANCA vasculitis was made. 

The Rheumatology service advised initiation of immunosuppression for possible salvage of renal function given that the patient’s creatinine had plateaued at 700 µmol/L; he became anuric and was initiated on intermittent hemodialysis one month following his admission to hospital. However, there is a paucity of data on the management of chronic inactive hepatitis B infection in patients who are on dialysis, limiting the provision of induction therapy with rituximab or cyclophosphamide in our patient. He was initiated on IV methylprednisolone 250 mg daily for 3 days, followed by an oral prednisone taper starting at 60 mg daily. This decision was made in conjunction with the Infectious Diseases service, given that he had sterilized his blood cultures roughly 1.5 weeks prior to steroid provision. The patient’s clinical course became complicated by development of pneumonia and candidemia, and he did not recover his renal function. The Cardiovascular (CV) surgery team advised against surgery given the patient’s complex medical status and comorbidities, and he died 3 months into his admission. 

## Literature review 

PubMed, Web of Science, Proceedings First, and Papers First databases were reviewed to look for similar cases of ANCA-associated GN in IE to determine treatment strategies, clinical manifestations, and prognosis. Search terms used to identify reports included “anti-neutrophil cytoplasmic antibody”, “glomerulonephritis”, and “endocarditis.” The articles reviewed were limited to those reporting biopsy-proven pauci-immune glomerulonephritis with the presence of antineutrophil cytoplasmic antibodies in patients with culture-positive infective endocarditis. Additionally, abstracts published from conference proceedings of the International Society of Nephrology were reviewed for similar case reports not published in peer-reviewed journals. 

Five additional cases of ANCA associated pauci-immune GN in patients with bacterial endocarditis were found (Table 1 – our case represented by A). All reported cases were male with ages ranging from 36 to 59 years of age. *Bartonella henselae* (B, F, E), *Streptococcus mutans* (C), and *Streptococcus oralis* (D) were isolated. Valvular involvement was limited to the aortic and mitral valves. When reported, the duration of the preceding infection varied from 1 (E) to 3 (B, C, F) months. All cases had detectable ANCA to PR3-antigen, and one also had detectable ANCA to the myeloperoxidase antigen (MPO, C). Extrarenal manifestations were variable including rash, arthralgias, and hemoptysis (B), and probable cerebral vasculitis (C). 

Four of the cases (B, C, E, F) had resolution of GN, while the other died immediately following CV surgery of postoperative cerebral hemorrhage (D). Three of the surviving patients received cyclophosphamide as initial treatment for their GN in addition to glucocorticoids (B, C, F), while 1 patient (E) received 2 doses of pulse methylprednisolone and subsequently completed a prednisone taper along with extended duration antibiotics. 

In the patients who underwent induction therapy, patient B received prednisolone for 12 weeks followed by azathioprine and prednisolone that was later switched to oral mycophenolate mofetil (MMF) 500 mg twice daily owing to complications of azathioprine. He was maintained on MMF and prednisolone for 9 months and maintained renal recovery. In contrast, patient C received oral cyclophosphamide and prednisone following induction. While the specific duration and dosing were not stated, both prednisone and cyclophosphamide were discontinued after one year due to resolution of symptoms including renal recovery and normalization of ANCA antibodies. Patient F was initially treated with cyclophosphamide and prednisone for an unspecified length of time prior to undergoing valvular replacement after which he was managed with antibiotics only. 

## Conclusion 

The development of GN during infection is associated with significant morbidity and mortality and diagnosis requires a high index of clinical suspicion. While antibiotics hasten renal recovery in immune-complex mediated GN in IE, ANCA-associated renal vasculitis often requires treatment with immunosuppression through induction with either cyclophosphamide or rituximab [9]. We report the challenging case of a man with PR3-ANCA positive pauci-immune GN secondary to IE whose concurrent viral hepatitides precluded provision of standard immunosuppressive strategies given the inability to treat his chronic inactive hepatitis B infection while on hemodialysis. Moreover, pathogenic autoantibodies including ANCAs may arise throughout the course of chronic hepatitis B and/or C infections, further complicating the underlying pathophysiology of our patient’s renal presentation. 

We found 5 additional cases of ANCA-associated GN in patients with IE, highlighting the rarity of this clinical scenario but also the need for a renal biopsy when unexplained kidney injury persists despite resolution of infection. Interestingly, all of the cases retrieved in our literature review were male. A large epidemiological study conducted in the UK revealed a slight predilection for males to develop ANCA vasculitis over females [10]; however, IE is more common in males making it unclear whether a gender predilection for ANCA-associated vasculitis in infection exists [11]. While all patients had ANCA directed against the PR3-antigen that is seen most often in granulomatosis with polyangiitis, none had upper airway disease or granulomas on biopsy, suggesting their disease might be best described as “PR3-associated ANCA vasculitis.” 

Development of ANCA-mediated GN occurred in subacute and chronic courses of infection, reflecting the need for prolonged antigenic stimulation in the development of ANCAs. Moreover, all reported cases except our own had left-sided endocardial involvement. Immunologic manifestations of IE are more common in left- vs. right-sided endocarditis, possibly owing to partial filtration of the antigenic load by the pulmonary vasculature in right-sided IE [12]. This may reduce the chronic immune stimulation that characterizes ANCA production in infection. Our patient had a high microbial burden given concurrent infection of his right-sided ICD lead, which may have explained the ability of ANCA-mediated vasculitis to develop. 

The majority of patients identified in our literature review recovered renal function and were treated with a combination of antibiotics and immunosuppression in the form of induction therapy with cyclophosphamide or pulse methylprednisolone and maintenance with azathioprine, MMF, prednisone, or antibiotics alone. Thus, the optimal strategy for immunosuppression in the management ANCA GN in IE is unclear and further complicated by the association between IE and pauci-immune GN independent of ANCA positivity, although exceedingly rare [3]. In patients with IE and ANCA-positive pauci-immune GN, the differentiation between ANCA-mediated and IE-mediated renal disease can be very challenging and requires clinical judgement. This will determine whether treatment with long-term immunosuppression in addition to infection is pursued. 

Ultimately, the role of ANCA in infection remains unclear, but the kidneys have proven to be a common, well-established target in disease states mediated by ANCAs. Persistent renal dysfunction in infectious diseases warrants exclusion of secondary development of autoimmune vasculitis affecting the kidneys through renal biopsy so as to not delay provision of organ-salvaging immunotherapy. 

## Conflict of interest 

No financial disclosures or conflicts of interest to declare. 

**Table 1.** Five cases of ANCA GN in the setting of infective endocarditis are described in the literature.

Patient demographics	Estimated duration of infection	Microbe	Cardiac involvement	Immunologic disturbances	Renal biopsy	AAV-related organ involvement	Treatment	Patient outcome	Reference
59 years, male (A)	6 months	*Enterococcus faecalis*	Tricuspid valve	PR3-ANCA C3 low C4 low	Focal segmental proliferative and necrotizing GN	Renal only – GN	Induction: pulse methylprednisolone Maintenance: prednisone	Patient died on post-admission day 75	Case patient
36 years, male (B)	3 months	*Bartonella henselae*	Aortic valve	PR3-ANCA	Pauci-immune necrotizing GN	Renal – GN; Skin – purpuric rash; MSK – arthralgia/myalgia; Respiratory – hemoptysis	Induction: prednisolone + IV cyclophosphamide Maintenance: Imuran, MMF, prednisone CV surgery	Resolved, on maintenance immunosuppression with MMF and prednisone only	[13]
53 years, male (C)	3 months	*Streptococcus mutans*	Mitral valve	PR3-ANCA MPO-ANCA RF positive C3 low	Pauci-immune GN + IN	Renal – GN and IN; CNS – cerebral vasculitis	Induction: IV cyclophosphamide Maintenance: prednisone	Resolved	[8]
50 years, male (D)	Unknown	*Streptococcus oralis*	Mitral valve	PR3-ANCA	Pauci-immune GN	Renal only – GN	Induction: IV steroids CV surgery	Patient died of brain hemorrhage post-op	[14]
55 years, male (E)	1 month	*Bartonella henselae*	Aortic valve Mitral valve	PR3-ANCA C3 low	Pauci-immune GN	Renal only – GN	Pulse methylprednisolone (× 2 days) + prednisone taper	Resolved with 6-month course of extended antibiotics	[15]
43 years, male (F)	3 months	*Bartonella henselae*	Bioprosthetic aortic valve, bioprosthetic mitral valve	PR3-ANCA	Pauci-immune GN	Renal only – GN	Cyclophosphamide + prednisone CV surgery	Resolved, chronic antibiotic therapy	[16]

CV = cardiovascular; IN = interstitial nephritis, GN = glomerulonephritis; MMF = mycophenolate mofetil.
